# Condensatopathies as a mechanistic framework for disease and integrated theranostic intervention

**DOI:** 10.7150/thno.127750

**Published:** 2026-01-01

**Authors:** Xin Li, Haiyan Wang, Jinghao Yao, Biwei Han, Xiaoxuan Zhao, Yuepeng Jiang, Huan Chen, Yan Yang, Hongwei Hou, Liang Wang

**Affiliations:** 1 Beijing Life Science Academy, Key Laboratory of Tobacco Biological Effects, Beijing, 102209, China.; 2 Phase Separation Biotechnology (Wuhan) Co., Ltd., Wuhan, 430075, China.; 3 Department of Medical Oncology, The First Affiliated Hospital of Bengbu Medical University, Bengbu, China.; 4 Key Laboratory of Pesticide & Chemical Biology of Ministry of Education, Hubei Key Laboratory of Genetic Regulation and Integrative Biology, School of Life Sciences, Central China Normal University, Wuhan, 430079, China.; 5 Department of Traditional Chinese Medicine (TCM) Gynecology, Hangzhou TCM Hospital Affiliated to Zhejiang Chinese Medical University, Hangzhou, 310007, China.; 6 College of Basic Medical Sciences, Zhejiang Chinese Medical University, Hangzhou, 310053, China.

**Keywords:** condensatopathy, biomolecular condensates, neurodegeneration, cancer, theranostics

## Abstract

The spatial organization of the cell relies on biomolecular condensates formed via liquid-liquid phase separation (LLPS). The dysregulation of this physicochemical order drives a growing class of human pathologies. Here, we champion the unifying term "Condensatopathies" and establish a rigorous framework for their classification based on three core criteria: genetic/environmental triggers, demonstrable biophysical defects, and causal toxicity. We synthesize the pathogenic landscape into two distinct yet interconnected mechanisms: Loss-of-Function (LOF), where essential condensates fail to form or harden; and Toxic Gain-of-Function (TGOF), characterized by the formation of aberrant, often solid-like aggregates or oncogenic hubs that hijack cellular machinery. By analyzing representative cases—from the biophysical maturation of TDP-43 in neurodegeneration to the chromatin hijacking by NUP98 fusions in leukemia—we reveal how the loss of "tunable metastability" underpins these disorders. Furthermore, we review how emerging technologies like optogenetics and cryo-ET are decoding these mechanisms. Finally, we propose an integrated "See-and-Treat" theranostic paradigm, utilizing the unique material properties of condensates to design specific diagnostic probes and "molecular scalpels" for precision intervention.

## 1. Introduction

A fundamental question in cell biology is how the densely packed cellular interior is organized to control complex biochemical reactions in space and time 1. While membrane-bound organelles provide one solution, for over a century, cell biologists have also recognized discrete, non-membranous bodies within the cell 2. Prime examples include the nucleolus and Cajal bodies, the latter first described as 'nucleolar accessory bodies' 3. These structures are now understood as dynamic hubs for ribonucleoprotein (RNP) metabolism, where key components are concentrated without a delimiting membrane 2, 4. The formation of Cajal bodies, driven by the phase separation of scaffold proteins like coilin, is a deeply conserved process essential for cellular quiescence 4. These structures, along with cytoplasmic RNP assemblies like germ granules, were long observed as enigmatic entities 5.

A conceptual breakthrough occurred with pioneering studies on *C. elegans* P-granules, which demonstrated that these germline bodies exhibit liquid-like properties, fundamentally linking them to the physical process of phase separation 6. This discovery, complemented by the seminal work showing that low complexity (LC) sequence domains from RNA-binding proteins can self-assemble into hydrogels composed of dynamic, amyloid-like fibers 7, reframed our understanding of these structures as "droplet organelles" 8. This principle is now central to understanding development and stress responses 9. The application of polymer physics revealed that these organelles are biomolecular condensates formed via liquid-liquid phase separation (LLPS), a concept that has reshaped modern cell biology 10. This framework posits that multivalent macromolecules can spontaneously demix from the surrounding nucleo- or cytoplasm into condensed liquid-like droplets 11. This phase transition is driven by a network of weak, transient, and often promiscuous interactions, which contrasts with the ordered, sequential assembly typical of stable multi-protein complexes like the ribosome 12. This spatial confinement serves distinct and vital physiological purposes. By concentrating enzymes and substrates within a discrete phase, condensates can act as reaction crucibles to accelerate biochemical rates. Conversely, they can sequester specific molecules to inhibit their activity or buffer cellular noise, ensuring precise spatiotemporal control over biological processes. The molecular drivers of LLPS are frequently proteins containing intrinsically disordered regions (IDRs) and nucleic acids 13. The convergence of research on intrinsically disordered proteins (IDPs) and membraneless organelles has provided a powerful mechanistic framework, positioning IDP-driven LLPS as a central mechanism for cellular organization 14. A key subclass of IDRs are prion-like domains (PrLDs), low-complexity sequences that are potent drivers of physiological LLPS but are also highly susceptible to pathogenic misfolding 15, 16. This "double-edged sword" nature was powerfully illustrated by a landmark 2013 study, which first demonstrated that mutations within the PrLDs of hnRNPA1 and hnRNPA2B1 are a direct cause of multisystem proteinopathy (MSP) and amyotrophic lateral sclerosis (ALS), and frontotemporal dementia (FTD) 17. These pathogenic mutations can accelerate aberrant liquid-to-solid phase transitions, converting functional condensates into the irreversible, amyloid-like fibrils seen in disease 18. However, PrLD-driven phase separation can also be a protective, adaptive behavior; for instance, the yeast prion protein Sup35 forms protective gels under stress to preserve its essential function 19. This delicate balance is actively maintained by a sophisticated network of molecular chaperones, which constantly survey and preserve the health of the proteome. Chaperones such as Hsp90 and the small heat shock protein HSPB1 (Hsp27) have been shown to directly target IDRs and aggregation-prone proteins like TDP-43 and Tau, highlighting that the maintenance of condensate fluidity is an active, chaperoned process 20, 21.

This organizing principle is universal, with multivalency emerging as a key feature across a vast array of biological contexts 22. It governs processes from mitotic spindle assembly 23 and synaptic organization 24 to the complex regulation of transcription 25. The dysregulation of these processes is central to a rapidly expanding list of human pathologies. Over the years, these conditions have been referred to by various names, including protein condensation diseases 26, aggregation disorders, and proteinopathies. While these terms have been invaluable, they primarily emphasize the end-stage formation of solid, often amyloid-like, protein aggregates. However, a growing body of evidence reveals that pathology can arise much earlier, from the subtle dysregulation of the liquid condensates themselves. To capture this broader mechanistic landscape, we adopt and formally define the unifying term—Condensatopathies. To ensure conceptual precision, we propose that classifying a disorder as a condensatopathy requires meeting three core criteria. Primarily, the pathology must be initiated by a distinct trigger, such as a genetic mutation or environmental insult, that directly modulates the phase separation propensity of a scaffold or client protein. Subsequently, this molecular alteration must manifest as a verifiable biophysical defect—whether it be an aberrant phase transition (e.g., from liquid to solid), a shift in composition, or erroneous spatiotemporal localization. Crucially, a causal link must be established, demonstrating that this biophysical aberration serves as the proximal driver of cellular dysfunction rather than acting merely as a secondary bystander. This definition encompasses not only primary defects in scaffold proteins but also pathologies driven by the dysregulation of upstream controllers (e.g., kinases or chaperones), provided that the proximal cause of cellular dysfunction is the aberrant state of the condensate.

The necessity for this expanded definition stems from the fact that the pathogenic event is not always a transition to a solid state. Condensatopathies encompass a wider spectrum of dysfunctions, including: (i) the failure of a functional condensate to form (a Loss-of-Function pathology), such as the inability to assemble DNA damage repair foci, leading to genomic instability; (ii) a shift in the material properties of a condensate, for instance, from a dynamic liquid to a viscous, gel-like state that impairs function without forming irreversible fibrils; and (iii) alterations in a condensate's composition or localization that hijack or sequester essential cellular components. Thus, unlike "proteinopathy," which focuses on a molecular endpoint (misfolded protein), "condensatopathy" pinpoints a disruption in a fundamental organizing principle of the cell (aberrant phase separation). This mechanistic framework links a vast array of diseases by their underlying physicochemical origin. A landmark bioinformatic study powerfully underscored this scope by associating over 36,000 pathogenic mutations from more than 1,200 Mendelian diseases and 550 cancers with the potential dysregulation of biomolecular condensates 27. This paradigm has already given rise to new disease classifications, such as “nuclear speckleopathies” for developmental disorders caused by variants in nuclear speckle proteins 28.

The pathogenic landscape of condensatopathies is now understood to be extraordinarily broad. It extends far beyond neurodegeneration and cancer to firmly include: cardiovascular diseases such as heart failure 29, dilated cardiomyopathy 30, and arterial stiffness 31; metabolic disorders including type 2 diabetes 32 and liver fibrosis 33; and numerous congenital defects, such as the complex malformation syndrome caused by aberrant HMGB1 phase separation 34. The list also encompasses sensory disorders like progressive hearing loss 35 and cataracts, reproductive disorders including male infertility, and psychiatric conditions such as epilepsy 36.

Furthermore, our understanding of the molecular architecture of these diseases is continuously being refined. Groundbreaking studies using cryo-electron microscopy (cryo-EM) on patient brain tissue have overturned dogma, revealing that the abundant amyloid filaments in FTLD-FUS are in fact composed of TAF15, establishing a new "TAF15 proteinopathy" 37. Similarly, an unbiased analysis of Parkinson's disease (PD) and Dementia with Lewy Bodies (DLB) brains uncovered novel nuclear inclusions composed of the RNA-binding proteins NONO/SFPQ and aberrantly edited mRNAs 38. These discoveries highlight the existence of a vast “dark matter” of condensatopathies yet to be explored and reveal complex cross-talk between proteinopathies, with evidence showing misfolded prion protein can directly cross-seed the aggregation and inactivation of TDP-43 39. Strikingly, recent evidence demonstrates that the same protein can give rise to distinct, tissue-specific condensatopathies; a frameshift variant of TDP-43 was found to cause a primary myopathy rather than classical ALS/FTD, underscoring the importance of cellular context 40. The list of triggers is also expanding to include environmental factors, with a recent study demonstrating that polystyrene nanoparticles can induce aberrant TDP-43 condensation and ALS-like symptoms 41, while physiological aging itself is increasingly viewed as a process driven by the progressive decline of MLO homeostasis 42.

This explosion in fundamental understanding has simultaneously unveiled a new frontier for clinical translation. The unique physicochemical properties of pathological condensates—their altered composition, material state, and localization—make them exquisitely specific biomarkers and therapeutic targets that are often absent in healthy cells 43, 44. This review provides a framework to understand these diseases by classifying their underlying pathogenic mechanisms: a Loss-of-Function (LOF) where physiological condensates are compromised, and a Toxic Gain-of-Function (TGOF) where aberrant condensates drive pathology. We will explore how these mechanisms often coexist in a "Double-Hit" paradigm, where the loss of nuclear function can, in a vicious cycle, promote the production of new aggregation-prone proteins through aberrant splicing 45. Critically, recent work has reconciled the seemingly contradictory roles of condensates through the concept of “tunable metastability,” explaining how they can act as both crucibles for and protectors against pathological aggregation 46. This is complemented by compelling *in vivo* evidence that condensates like stress granules are primarily protective, and that their formation failure represents a key LOF mechanism in neurodegeneration 47. By bridging the fundamental physics of phase separation with the pathology of human disease, and contextualizing these events within the broader decline of cellular homeostasis during aging 48, 49, we can begin to design integrated "see-and-treat" theranostic strategies. These strategies, often involving "condensate modifying drugs" (c-mods) 44, are not limited to correcting pathological condensation but extend to harnessing the principles of LLPS to engineer more effective cellular therapies, such as next-generation Chimeric Antigen Receptor (CAR)-T cells 50. The ultimate goal is to visualize, modulate, and ultimately restore condensate homeostasis for therapeutic benefit, leveraging the finding that protein mislocalization is a pervasive mechanism underlying a vast array of human disorders 51.

## 2. The Physicochemical Grammar of Condensate Homeostasis

The formation, function, and dissolution of condensates are governed by a precise "molecular grammar" encoded in the sequence and structure of multivalent macromolecules (Table 1). This grammar is exquisitely sensitive to the cellular environment, with a complex interplay between molecular features and environmental cues determining the final state 52. Each element of this grammar represents a potential point for diagnostic sensing or therapeutic intervention.

### 2.1 Proteins and the Stickers-and-Spacers Model

**Figure 1:**
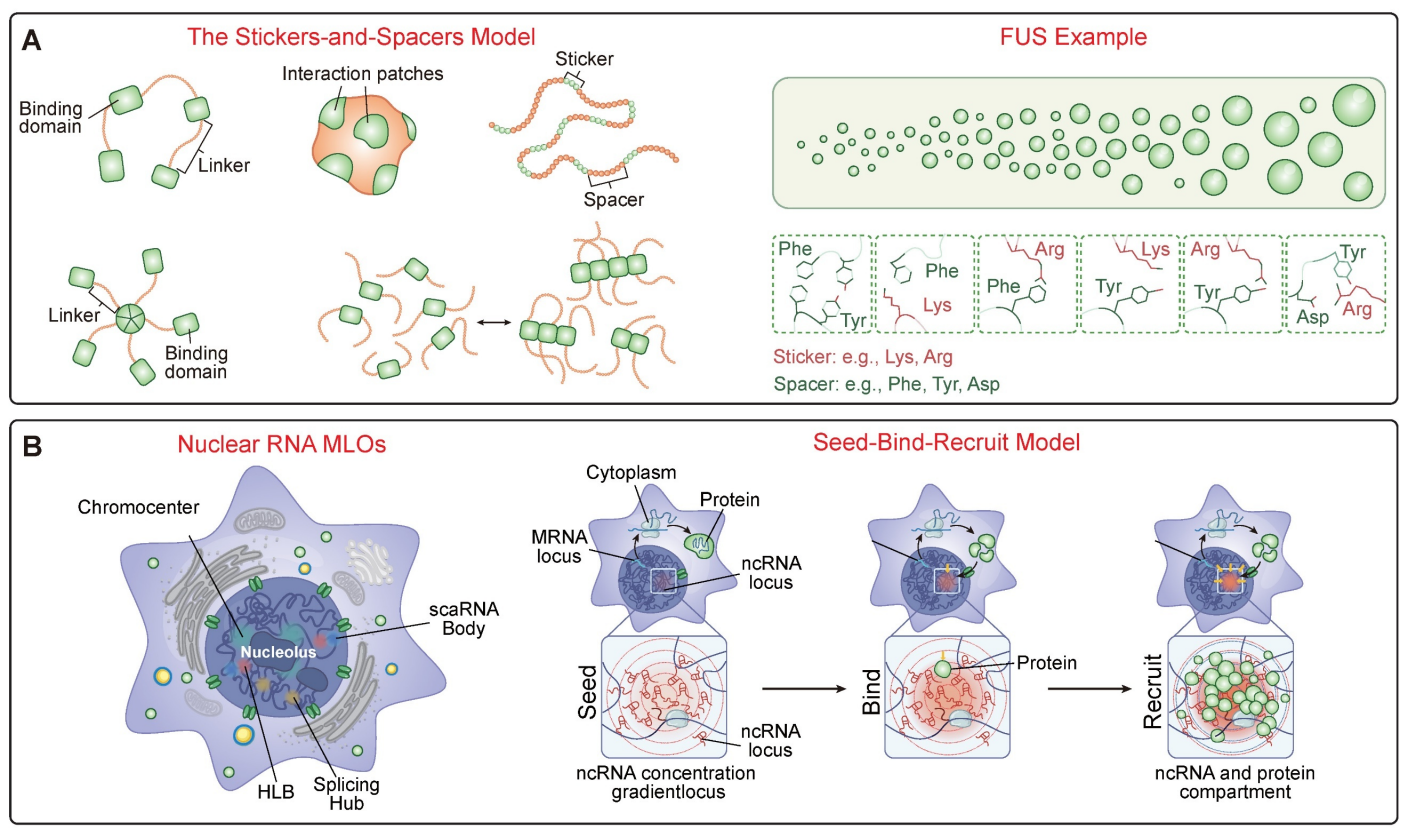
** The Molecular Grammar of Biomolecular Condensates.** (A) The "Stickers-and-Spacers" Architecture. This panel illustrates the fundamental protein grammar governing phase separation. The schematic (Left) depicts the model where "stickers" are motifs mediating attractive interactions (e.g., folded domains, specific residues) and "spacers" are flexible linkers regulating material properties. The FUS protein (Right) exemplifies this predictive grammar, where the phase transition from a diffuse state to liquid droplets is driven by multivalent interactions between specific sticker residues, such as Tyrosines in the Prion-like Domain (PrLD) and Arginines in the RGG domain. (B) RNA-Mediated Compartmentalization. RNA functions as a multivalent scaffold to organize nuclear architecture. The schematic illustrates the "Seed -> Bind -> Recruit" mechanism: non-coding RNA (ncRNA) transcribed at a genetic locus acts as a high-concentration "Seed." This seed then "Binds" diffusible regulator proteins, subsequently "Recruiting" additional clients to assemble a concentrated, functional compartment (e.g., nucleolus or splicing hub) at the specific genomic site.

The behavior of condensate-forming proteins is well-described by the “stickers-and-spacers” model 22 (Figure 1A). “Stickers” are discrete regions (e.g., aromatic residues like tyrosine, charged motifs like arginine, folded domains, or coiled-coils) that mediate attractive interactions, while “spacers” are the intervening sequences that modulate condensate properties like flexibility and viscosity 53. A systematic dissection of the FUS protein has revealed a predictive molecular grammar where multivalent interactions between tyrosine residues in the prion-like domain and arginine residues in the RNA-binding domain act as the primary stickers that govern the saturation concentration for phase separation. The material properties of the resulting condensate are tuned by the spacers: glycine residues enhance fluidity and maintain a liquid-like state, whereas glutamine and serine residues promote hardening into less dynamic, gel-like states 54. This framework is now being used to model pathologies, where missense mutations associated with various neurological diseases often manifest their effect by enhancing the stability of otherwise labile molecular structures formed upon the self-association of low-complexity domains (LCDs) 55. For example, the toxicity of arginine-rich dipeptide repeats (DPRs) produced from the *C9orf72* gene is determined not just by the sheer number of arginine "stickers," but by the biochemical properties and specific alternating distribution pattern of the spacer amino acids, which dictates how they interact with cellular partners and drive aberrant phase separation 56.

A prime example of repressive condensate formation, where chromatin is an active, compacted participant 57, is the multivalent engagement of histone modifications. A foundational study demonstrated that the formation of constitutive heterochromatin is driven by a writer-reader system that triggers LLPS. Complexes containing the H3K9 methyltransferase SUV39H1 (writer), the reader protein HP1 (reader), and the scaffold TRIM28 possess multiple chromodomains that act as “stickers.” These multivalently engage H3K9me3-marked nucleosomes, creating a network of interactions sufficient to drive the phase separation of chromatin into dense, liquid-like droplets that exclude transcriptional machinery 58. This process is further regulated by the dynamic competition and interplay between different Chromatin-Associated Proteins (CAPs). The transcriptional repressor MeCP2, for instance, compacts chromatin through phase separation, a process that is compromised by mutations causing the neurodevelopmental disorder Rett syndrome. In the nucleus, MeCP2 and the linker histone H1 form distinct, mutually exclusive condensates, competing for chromatin occupancy. RTT-causing mutations weaken MeCP2's phase separation capacity, disrupting this balance and allowing H1-rich condensates to dominate the heterochromatic landscape 59. H1's ability to drive condensation is itself a conserved mechanism, mediated by its C-terminal intrinsically disordered region 60. The regulation of condensate assembly is made even more complex by accessory proteins that can promote phase separation of primary scaffolds (e.g., coiled-coil proteins in plants 61) and by non-canonical drivers, such as histone variants with unique IDRs 62.

This intrinsic grammar is not static; it is constantly being read and modulated by extrinsic factors like molecular chaperones. A prime example is the Hsp90 chaperone, which acts as a master governor of IDR-containing proteins. A chemical-biology approach revealed that Hsp90 physically associates with a large fraction (~20%) of the yeast proteome, preferentially targeting and binding IDRs to maintain their solubility and prevent their spontaneous transition into stress granules or P-bodies at physiological temperatures 20. Other chaperones, such as Hsp70 and Hsp40, can themselves undergo phase separation to efficiently enter and regulate condensates from within 63. This extends to non-canonical chaperones; for instance, the Hippo pathway effector YAP maintains the dynamics of TDP-43 condensates 64, and β-synuclein co-condenses with α-synuclein to block its pathological maturation 65.

### 2.2 RNA as a Master Regulator

RNA is not a passive client but an active and potent regulator of condensate assembly 66. A foundational concept is that non-coding RNAs (ncRNAs) can act as scaffolds to organize nuclear compartments through a "Seed -> Bind -> Recruit" mechanism (Figure 1B). In this model, the act of transcription creates a high local concentration of an ncRNA near its genetic locus, which acts as a "seed." This high concentration of RNA then "binds" diffusible regulatory proteins and other ncRNAs through high-affinity interactions. This binding, in turn, "recruits" these factors into a spatially confined territory, driving the formation of a functional compartment. This mechanism helps explain the formation of numerous nuclear bodies associated with RNA processing, heterochromatin assembly, and gene regulation 67. A foundational concept in this regulation is the phenomenon of re-entrant phase transition, where RNA can both promote and inhibit condensate formation depending on its concentration relative to RNA-binding proteins (RBPs) 68. At sub-stoichiometric ratios, RNA acts as a multivalent scaffold, promoting LLPS by creating a network of interactions that can achieve charge balance. However, at super-stoichiometric ratios, each RNA molecule becomes coated with RBPs, leading to charge imbalance and net repulsion between the soluble complexes, which in turn leads to condensate dissolution 69. This provides a powerful self-regulation mechanism. For instance, in transcriptional hubs, the low levels of nascent RNA produced during transcription initiation can stimulate condensate formation, while the subsequent high levels of mRNA produced during a transcriptional burst can create a negative feedback loop that automatically dissolves the condensate, thereby terminating the burst of gene expression 70.

Beyond concentration, RNA possesses an intrinsic ability to self-assemble and can undergo a networking transition known as percolation, creating stable, gel- or solid-like networks 71. This "RNA code" is critical in pathology, as disease-relevant repeats like (GGGGCC)n are prone to forming G-quadruplex structures that drive these stable condensates 72. Remarkably, this is not limited to long repeats; even short RNAs with just two repeats can form solid-like foci if they adopt G4 structures 73. Conversely, RNA can also be protective, as high concentrations can maintain the fluidity of RBP condensates and prevent their pathological liquid-to-solid transition 74. Indeed, its importance is so fundamental that its enzymatic degradation in cell lysates leads to the widespread aggregation of over 1,300 proteins, suggesting RNA acts as a broad molecular chaperone 75. Post-transcriptional modifications add another layer of control; N6-methyladenosine (m6A) acts as a molecular switch read by YTHDF proteins to scaffold stress granule formation 76, while a newly discovered modification, N1-methyladenosine (m1A) on CAG repeat RNA, can directly bind TDP-43 and induce its pathology 77. Long non-coding RNAs (lncRNAs) also play a crucial role as scaffolds. For instance, *MALAT1* is essential for the assembly of nuclear splicing speckles, and its upregulation in metastatic cancers drives aberrant splicing programs 67. Other scaffolds include NEAT1 for paraspeckles, NORAD which sequesters PUMILIO proteins 78, and various oncogenic lncRNAs that drive the assembly of cancer-promoting condensates.

### 2.3 Active Droplets, Material Properties, and Environmental Integration

The molecular grammar dictates the material properties of condensates, such as viscosity and surface tension 79. Many are "active droplets," maintained in a non-equilibrium state by energy-consuming enzymes like ATP-dependent helicases and kinases, which allows them to resist pathological solidification and perform work 80. The nucleolus is a prime example of a complex, multiphase active condensate, whose layered architecture spatially organizes the intricate steps of ribosome biogenesis 81.

**Figure 2:**
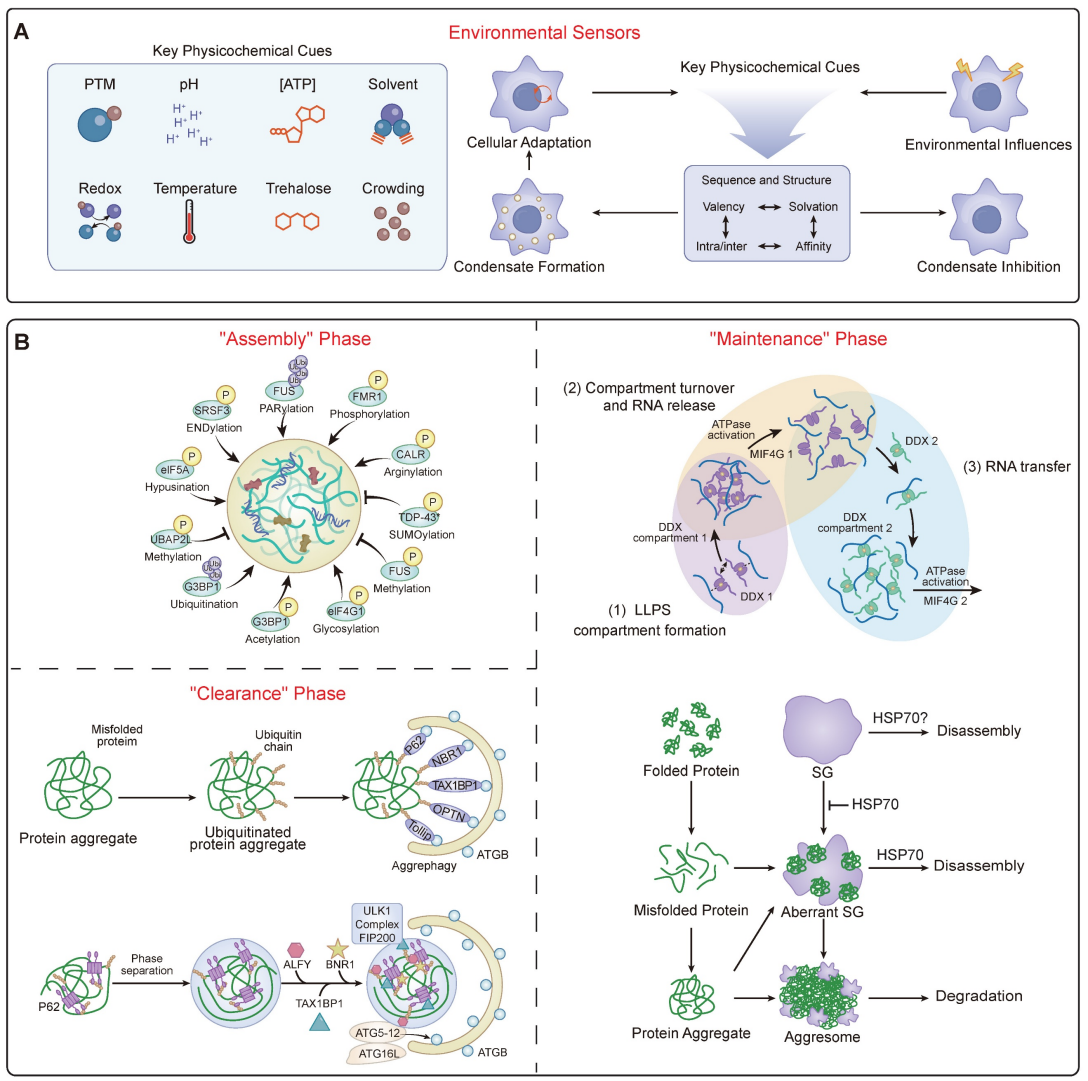
** The Dynamic Landscape of Condensate Homeostasis.** (A) Environmental Integration. This panel summarizes how the regulatory machinery acts as a sensor for physicochemical cues. It illustrates how diverse signals serve as input signals to modulate the liquidity and stability of the condensate, ensuring adaptive cellular responses to stress. (B) The Dynamic Regulatory Cycle. A schematic flowchart responding to the need for mechanistic clarity. It illustrates the continuous lifecycle of a condensate, maintained in a non-equilibrium state ("Granulostasis"). The cycle depicts the "Assembly" phase driven by PTM codes (e.g., phosphorylation); the "Maintenance" phase, where ATP-dependent enzymes (e.g., DDX helicases) and chaperones (e.g., HSP70) actively expend energy to remodel RNP networks and prevent solidification; and the "Clearance" phase, where terminally damaged condensates are recognized by receptors like p62/SQSTM1 and removed via selective autophagy ("granulophagy").

Beyond their intrinsic activity, condensates are sophisticated environmental sensors, capable of integrating a wide array of intracellular and extracellular signals 48 (Figure 2A). Their assembly and properties are exquisitely sensitive to the physicochemical milieu, responding to shifts in temperature via thermosensors like HSF1 82, changes in pH through histidine-rich domains 83, and alterations in redox state via methionine oxidation 84. This environmental sensing is a critical adaptive mechanism. For instance, cell shrinkage due to hypertonic stress increases cytosolic macromolecular crowding, a physical change that is directly sensed by WNK kinases. This crowding triggers WNK1 to phase separate into functional condensates that activate a signaling cascade to restore cell volume, demonstrating how a physical stress is translated into a biochemical response via phase separation 85.

Cellular ionic strength is another critical regulator. Under hyperosmotic stress, the cation channel TRPM4 facilitates a rapid influx of sodium ions (Na⁺). This influx is not just for volume recovery; it directly modulates the liquidity of multiple stress-induced condensates, including those formed by ASK3 and TAZ. The elevated intracellular Na⁺ acts to shield electrostatic interactions, preventing the pathological liquid-to-solid transition of these condensates and thereby maintaining their function during the stress response 86. Beyond specific ions like Zn²⁺ gating assembly 87, this highlights a broader principle where fluxes of common ions like Na⁺ directly tune the material state of condensates. Furthermore, condensates are integrated with the cell's physical architecture, with their assembly modulated by interactions with membranes 88 and the cytoskeleton, and even driven by external mechanical forces like matrix stiffness, which can trigger oncogenic phase separation 89. Cellular energy status is paramount, as ATP acts as a crucial biological hydrotrope whose depletion promotes pathological aggregation of neurodegenerative proteins in mammalian neurons 90, 91. This environmental integration implies that disease states associated with altered cellular physiology—be it ionic imbalance, oxidative stress, or metabolic shifts—can directly trigger condensatopathies.

### 2.4 Post-Translational Regulation and Protein Quality Control

A primary layer of regulation is provided by a vast repertoire of PTMs, which act as a master regulatory code for condensate dynamics (Table 2) (Figure 2B). These modifications can either promote or inhibit LLPS and subsequent phase transitions. Phosphorylation is a particularly sophisticated switch, capable of both promoting and dissolving condensates; this is exemplified by the RNA Polymerase II CTD and globally managed by kinases like DYRK3, which acts as a master “dissolvase” for multiple MLOs during mitosis 92. The ubiquitin and SUMOylation systems are also central, not only marking proteins for degradation but also signaling for condensate assembly and disassembly through recruitment of segregases like p97/VCP or regulating the dynamics of disassembly-engaged proteins 93. A diverse array of other PTMs, including arginine methylation, PARylation, acetylation, glutathionylation, and nutrient-sensing modifications like O-GlcNAcylation and lactylation, provide distinct layers of control that intimately link cellular signaling and metabolic states to condensate homeostasis 94. For example, PARylation and acetylation of ALS-associated proteins tend to promote phase separation, whereas phosphorylation, methylation, and citrullination are generally inhibitory 95.

To prevent pathological maturation and maintain this homeostasis, cells employ robust protein quality control (PQC) systems in a process termed 'granulostasis' 49 (Figure 2B). This PQC network is multi-layered. It includes RNA chaperones like ATP-dependent DDX helicases that actively remodel and resolve gel-like RNA networks using the energy from ATP hydrolysis, thereby controlling RNA flux into and out of condensates and maintaining their fluidity 80. It also includes a host of protein chaperones such as Hsp70, Hsp90, and small heat shock proteins (sHSPs) that directly engage with condensate components to maintain their liquidity and prevent aggregation 20, 21, 96. When these frontline systems are overwhelmed, cells utilize specialized PQC hubs. The nucleolus, for instance, acts as a processing center by sequestering misfolded proteins into its liquid-like granular component, which prevents their irreversible aggregation and keeps them competent for Hsp70-dependent refolding 97. PML bodies serve a similar quality control function 98. The cell's PQC arsenal is still expanding, with the recent discovery of novel compartments like ubiquitin-independent BAG2 condensates for proteasomal targeting 99, and specialized DHX9-SGs that specifically compartmentalize damaged RNA 100. For terminally damaged condensates, selective autophagy (granulophagy) serves as a final clearance mechanism, often mediated by receptors like p62/SQSTM1 that recognize and target condensates for degradation 101. The failure of these multi-layered PQC systems, particularly during aging, is a key driver of disease 48.

### 2.5 Technological Frontiers: Deciphering the Condensate Code

Our understanding of condensates has been revolutionized by a suite of emerging technologies that allow us to dissect their properties with unprecedented resolution. To establish causality, optogenetic systems such as "OptoDroplets" utilize light-sensitive domains (e.g., Cry2) fused to IDRs, enabling researchers to trigger phase separation with precise spatiotemporal control. This approach has been instrumental in demonstrating that the act of condensation itself—independent of protein expression levels—can drive signaling or toxicity 10, 102. Complementing this functional control is *in situ* cryo-electron tomography (cryo-ET), which is now bridging the resolution gap by revealing the molecular sociology within condensates. Unlike fluorescence imaging, cryo-ET captures structural details at the nanometer scale, allowing researchers to distinguish between amorphous liquid phases and ordered amyloid fibrils within native cells, a distinction critical for designing structure-based inhibitors 18. Furthermore, to quantify material properties, microrheology techniques and novel sensors are being employed. Tools such as passive particle tracking and viscosity-sensitive molecular rotors allow for the quantitative mapping of intracellular viscosity, revealing how "aging" condensates harden over time, which serves as a key biomarker for early-stage disease 103. Despite challenges such as the potential phototoxicity of optogenetics and the low throughput of cryo-ET, the integration of these tools is shifting the field from phenomenology to quantitative biophysics.

## 3. Pathogenic Mechanisms of Condensatopathies: Synthesis of LOF and TGOF

Disease arises when the homeostatic control of condensates is lost. While the phenotypic manifestations are diverse, the underlying biophysical mechanisms can be synthesized into two primary categories: LOF and TGOF. Despite their opposing outcomes, both mechanisms stem from a shared failure of "tunable metastability"—the inability of the cell to maintain proteins in their optimal, functional phase. In LOF pathologies, the energy barrier for assembly is often too high, preventing formation, or conversely, the condensate becomes overly rigid, impeding necessary internal dynamics. In contrast, TGOF pathologies occur when the barrier to aggregation is too low, leading to the formation of thermodynamically stable but toxic structures. Crucially, these mechanisms are not mutually exclusive; a single pathogenic event often triggers both, as the sequestration of a protein into a TGOF aggregate inevitably leads to its functional depletion (LOF) in the soluble pool. However, distinguishing the dominant driver is critical for therapeutic design: LOF diseases generally require "recruiters" or "plasticizers" to restore formation and liquidity, whereas TGOF diseases demand "inhibitors" or "degraders" to dissolve or remove the aberrant phase.

This disruption of metastability can originate from genetic mutations that directly alter the assembling components, from the dysregulation of key regulators (e.g., kinases or chaperones), or from global shifts in the cellular physicochemical environment 104. Arguably the most profound and universal of these shifts is physiological aging. While specific mutations can trigger disease at any age, aging acts as a master catalyst that creates a permissive environment for pathology to emerge and progress. The age-dependent vulnerability to condensatopathies is not a coincidence but a direct consequence of a vicious cycle involving both passive decay and active reinforcement of the senescent state.

On one hand, aging promotes pathology through the passive decline of cellular resilience. Key PQC systems, responsible for maintaining "granulostasis," progressively lose efficiency. The capacity of molecular chaperones like Hsp90 and Hsp70 diminishes, and clearance pathways like autophagy become impaired, leading to the accumulation of misfolded proteins 20, 49. This is compounded by metabolic dysregulation, notably a reduction in ATP, a crucial biological hydrotrope whose depletion lowers the energetic barrier for pathological aggregation 90, 91. This systemic decline systematically dismantles the safeguards of condensate homeostasis, making cells more susceptible to both genetic predispositions and stochastic insults.

On the other hand, aging is not merely a passive decay but also involves the active, gain-of-function assembly of specific condensates that reinforce the senescent state. A landmark study revealed that the protein SGF29 forms distinct nuclear condensates specifically in senescent cells. These are not inert byproducts but functional hubs that recruit transcriptional machinery to actively drive the expression of pro-senescence genes like *CDKN1A* (p21), effectively locking the cell into an aged state 105.

**Figure 3:**
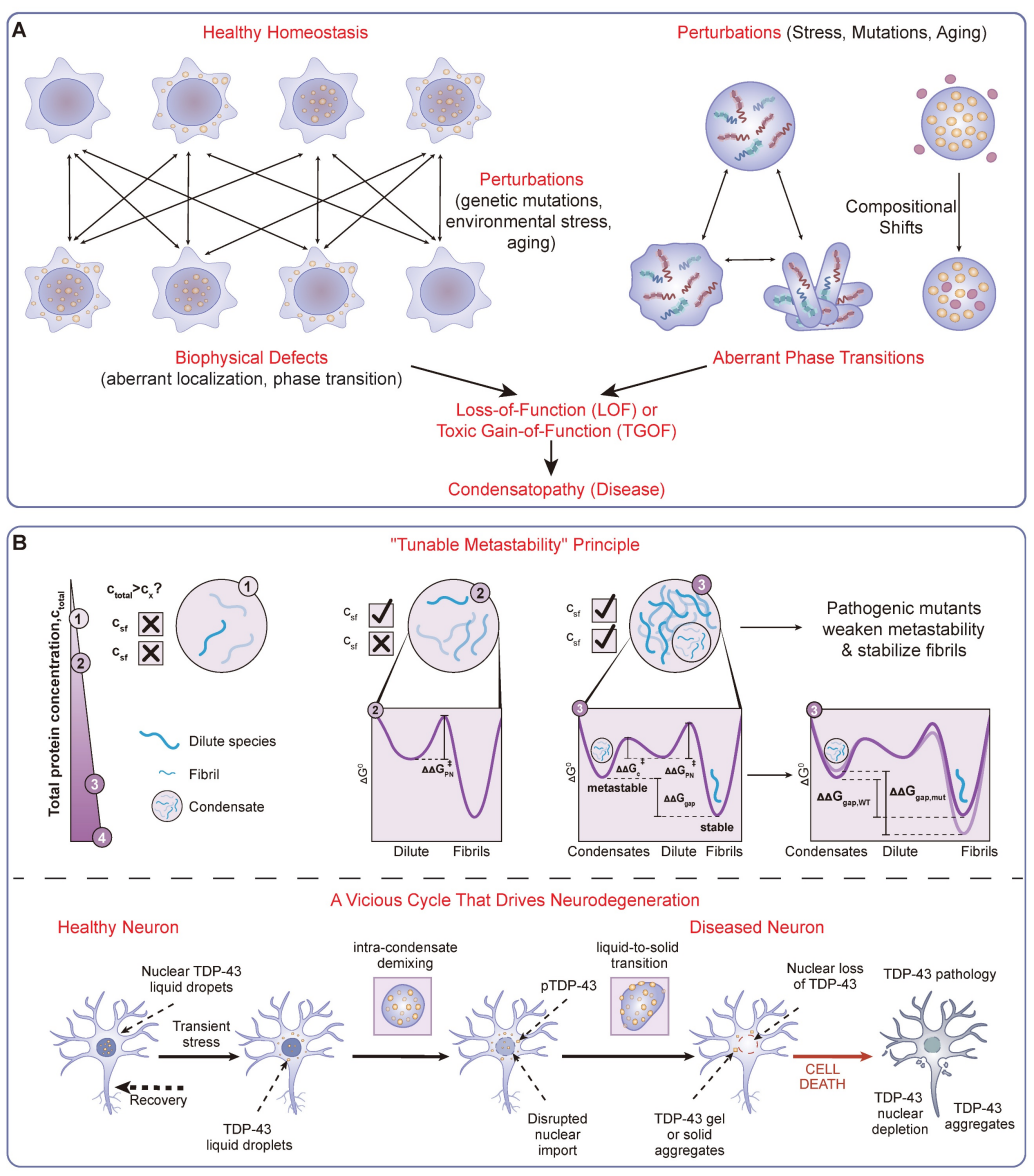
** A Mechanistic Framework of Condensatopathies.** (A) Classification Framework. A logic flowchart delineating the definition and categorization of condensatopathies. It traces the pathological trajectory from "Triggers" (genetic mutations, environmental stress, aging) to specific downstream alterations, manifesting as either aberrant phase transitions (e.g., liquid-to-solid) or compositional shifts. These defects branch into two core mechanisms: Loss-of-Function (LOF), characterized by failed assembly or functional deficiency; and Toxic Gain-of-Function (TGOF), characterized by aberrant aggregation or sequestration. (B) The "Double-Hit" Pathogenic Timeline. A temporal schematic illustrating the interplay between TGOF and LOF mechanisms, exemplified by TDP-43 proteinopathy. The timeline shows the progression from a metastable liquid state to a pathological "Liquid-to-Solid Transition" (LST), driven by the "Tunable Metastability" principle. This results in cytoplasmic aggregation (TGOF), which sequesters nuclear transport factors and consequently leads to nuclear depletion and loss of splicing function (LOF), forming a vicious cycle that drives neurodegeneration.

Therefore, aging constitutes a central, multifaceted mechanism that simultaneously erodes protective systems (a Loss-of-Function of PQC and metabolic stability) while actively building pro-aging pathological structures (a Toxic Gain-of-Function). This dual impact provides a critical context for understanding the origins of condensatopathies. The resulting perturbations lead to specific biophysical consequences—such as altered saturation concentrations, aberrant liquid-to-solid transitions, and ectopic condensate formation (Figure 3A)—which can be mechanistically classified as LOF, TGOF, or a "Double-Hit" combination. A landmark bioinformatic analysis underscored the immense scope of this problem, linking over 36,000 pathogenic mutations to the plausible dysregulation of biomolecular condensates 27. Understanding these mechanisms provides a clear framework for identifying vulnerabilities for diagnostic and therapeutic intervention.

### 3.1 Loss-of-Function (LOF): Failure to Form or Function

In LOF condensatopathies, disease results from the failure of physiological condensates to assemble or function correctly (Figure 3A). This failure itself can be a diagnostic indicator. A prime example is an impaired DNA Damage Response. The formation of transient repair compartments at sites of DNA damage is critical for genome integrity, a process driven by the LLPS of key factors like PARP1, FUS, and the MRNIP/MRN complex 106-108. When these proteins fail to form condensates, the efficient recruitment of repair machinery is compromised, leading to genome instability. Similarly, the tumor suppressor 53BP1 regulates heterochromatin stability through LLPS with HP1α; its loss leads to heterochromatin defects and cellular senescence 109, 110.

Developmental and congenital disorders represent another major class of LOF condensatopathies, as the precise spatiotemporal assembly of condensates is critical for these processes. For example, mutations in *SMN1* cause Spinal Muscular Atrophy by disrupting the SMN complex's ability to phase separate and assemble spliceosomes within Cajal bodies 111. This principle extends to a wide array of developmental disorders, including neurofibromatosis type 2 (*NF2* mutations disrupt LLPS with Hippo pathway components) 112, Wiskott-Aldrich syndrome (mutant WASP fails to form nuclear condensates that regulate splicing) 113, and a spectrum of ciliopathies, hearing loss, and myasthenic syndromes where mutations in key scaffold proteins impair the formation of essential condensates like the DynAPs at the ciliary root, tip-link densities in the inner ear, or the neuromuscular junction 114-116.

Rett syndrome (RTT) provides a canonical example of a condensatopathy where disease-causing mutations directly impair phase separation, leading to a loss of nuclear organization. The disease is caused by mutations in MeCP2, a crucial chromatin organizer that binds methylated DNA. A key study demonstrated that MeCP2 induces chromatin compaction by triggering the LLPS of nucleosomal arrays into dense condensates. This process is essential for organizing heterochromatin architecture. Crucially, the investigation revealed that a wide range of RTT-causing missense and nonsense mutations, particularly those in the methyl-binding (MBD) and transcriptional repression (TRD) domains, directly compromise MeCP2's ability to drive chromatin phase separation. This represents a clear LOF mechanism at the biophysical level. This functional impairment also disrupts MeCP2's ability to compete with linker histone H1, which forms its own distinct condensates. In cells with RTT mutations, MeCP2 fails to form its characteristic foci and is displaced from chromocenters, which instead become occupied by H1. This work establishes a direct mechanistic link from genetic mutation to aberrant phase separation and downstream pathological changes in chromatin organization, defining RTT as a disease of dysfunctional condensate formation 59.

Finally, a crucial LOF mechanism is the failure to form protective condensates. Compelling *in vivo* evidence now shows that specifically inhibiting stress granule (SG) formation in various neurodegenerative disease models exacerbates pathology, highlighting the fundamentally protective role of SGs 47. Indeed, *in vivo* studies on mutant TDP-43 ALS mice have shown that their central nervous system is deficient in SG assembly, supporting the idea that the inability to mount a proper stress response is itself pathogenic 117. This protective function may stem from the ability of SGs to sequester executioner caspases, thereby inhibiting apoptosis 118, or to relieve the burden on the nuclear PQC system by sequestering misfolded cytosolic proteins 119. The age-dependent decline in the ability to form SGs may therefore be a key contributor to age-related vulnerability 120.

### 3.2 Toxic Gain-of-Function (TGOF): Aberrant Assemblies, Sequestration, and Hijacking

TGOF pathologies are driven by the formation of abnormal condensates that are directly toxic, sequester essential cellular components, or hijack cellular machinery. These aberrant structures are highly specific disease markers and ideal therapeutic targets.

A primary TGOF mechanism is the pathological maturation of condensates, often involving a liquid-to-solid transition (LST) into irreversible, amyloid-like aggregates—a central event in neurodegeneration 121, 122. The governing principle for this transition is the concept of “tunable metastability” 46 (Figure 3B). Healthy cells invest energy to maintain proteins within a shallow, metastable liquid energy well. Pathogenic mutations or stress can lower the barrier, allowing proteins to fall into a deeper, thermodynamically stable solid state. Foundational studies on ALS-linked FUS and Tau have provided evidence for this accelerated transition 18, 123.

This phenomenon is best exemplified by TDP-43. Structural studies suggest that a hydrophobic alpha-helical region within its disordered C-terminal domain acts as a key "sticker." Disease-causing mutations often stabilize this helix, increasing intermolecular affinity. This subtle structural shift drastically alters the phase behavior, accelerating the maturation of liquid droplets into solid, amyloid-like fibrils that are resistant to clearance, thereby identifying the helix as a precise drug target 84.

A second major TGOF mechanism involves the sequestration of essential cellular components or the hijacking of cellular machinery by aberrant condensates. In repeat expansion disorders, this process can be driven by the RNA itself. For instance, (GGGGCC)n repeats in C9orf72-ALS/FTD form G-quadruplex structures that drive a percolation transition into stable, gel-like nuclear foci that sequester RBPs and are toxic 72, 124.

In cancer, TGOF is frequently driven by the formation of "oncogenic condensates," which act as neomorphic hubs that rewire the cell's transcriptional and signaling landscape. In NUP98-HOXA9 driven leukemia, the mechanism is a specific hijacking of the "stickers-and-spacers" grammar. The NUP98 IDR contains multiple FG (Phenylalanine-Glycine) repeats. Upon fusion with the DNA-binding domain of HOXA9, these FG repeats—which normally facilitate transport in the nuclear pore—act as potent stickers that recruit the transcriptional co-activator p300 via hydrophobic interactions. This creates a high-density "super-enhancer-like" condensate that physically traps the chromatin machinery, driving the overexpression of oncogenes like *MEIS1* 125. These oncogenic condensates represent a distinct pathological state, creating novel, spatially confined reaction crucibles for oncogenic processes. Similarly, in synovial sarcoma, the SS18-SSX fusion protein utilizes its IDR to displace polycomb components and form aberrant condensates on chromatin, hijacking the BAF complex 126.

Viruses have also evolved sophisticated TGOF mechanisms to hijack host LLPS machinery. The SARS-CoV-2 Nucleocapsid (N) protein, for instance, leverages its multivalency to phase separate with viral RNA, forming replication hubs while simultaneously infiltrating and dissolving host stress granules to blunt the innate immune response 127, 128.

### 3.3 The "Double-Hit" Paradigm: Coexistence of LOF and TGOF

In many condensatopathies, particularly neurodegenerative diseases, LOF and TGOF mechanisms are inextricably linked in a devastating vicious cycle (Figure 3B). This toxic interplay often unfolds within the context of an aging cellular environment. It is precisely the age-related decline in the capacity of PQC systems and the impairment of nucleocytoplasmic transport that provides the fertile ground for these pathologies to emerge.

In this context, TDP-43 proteinopathy serves as the canonical example, unfolding in a clear sequential cascade: initial cytoplasmic aggregation (TGOF) leads to nuclear depletion, which in turn causes a catastrophic loss of essential splicing functions (LOF) 129. This process can be triggered by external amyloid-like fibril "seeds," which induce a cascade that recapitulates both key disease hallmarks 130. The cytoplasmic de-mixing of TDP-43 disrupts nucleocytoplasmic transport, in part by recruiting critical import machinery into the aberrant condensates. This impairment of nuclear import exacerbates the depletion of nuclear TDP-43, creating a vicious cycle that ultimately leads to cell death 129. This nuclear LOF has tangible molecular consequences, leading to a unique transcriptomic signature characterized by RNA splicing defects, including the inclusion of disease-specific cryptic exons 130. This interplay is not limited to a single protein. In *C9orf72*-ALS/FTD, the toxic poly-PR dipeptides (TGOF) can interact with and disrupt the normal phase behavior of FUS, impairing its DNA damage repair function (LOF) 131. Similarly, in Parkinson's disease, the pathological accumulation of α-synuclein (TGOF) aberrantly interacts with and disrupts the function of P-body condensates, leading to defects in mRNA stability (LOF) 132. Understanding this interplay is essential for designing effective therapies that must address both the toxic aggregates and the functional deficits they cause.

## 4. Theranostic Strategies for Condensatopathies: A New Frontier in Precision Medicine

The mechanistic framework of condensatopathies does not merely classify diseases; it provides a rational basis for designing novel theranostic strategies aimed at restoring homeostasis 26, 43 (Table 4). This emerging paradigm moves beyond traditional pharmacology—which typically targets the active sites of well-folded enzymes—to address the physical state, composition, and organization of macromolecules. It opens up unprecedented opportunities for a truly integrated clinical approach, where the ability to visualize and quantify aberrant condensates directly informs and guides their therapeutic modulation. The growing arsenal of "condensate-modifying drugs" (c-mods) heralds this new approach, but their optimal use hinges on a tightly coupled diagnostic-therapeutic loop 44.

### 4.1 The Integrated Theranostic Loop: A See-and-Treat Paradigm

A core tenet of theranostics is the seamless integration of diagnosis and therapy, creating a dynamic feedback loop that enables personalized and adaptive treatment. For condensatopathies, this process can be conceptualized as an integrated cycle beginning with the ability to "See"—to non-invasively detect and characterize pathological condensates in patients. This extends beyond simple detection to quantifying specific biophysical properties like viscosity or polarity in real-time, a frontier being advanced by tools like fluorogenic sensors and molecular rotors that selectively illuminate solid-like aggregates of proteins such as Tau or α-synuclein 103, 133. On a broader scale, LLPS-related gene expression signatures are also emerging as powerful prognostic biomarkers 134, 135. Based on this detailed diagnostic data, clinicians can then "Decide" on a precise therapeutic strategy. For instance, a patient with early-stage, liquid-like oncogenic condensates might receive drugs to disrupt their formation, whereas a patient with late-stage, solid amyloid inclusions would require molecules designed to dissolve or clear them. This decision-making process directly links the physical pathology to a tailored molecular intervention, which is then administered in the "Treat" phase. This could involve a range of c-mods, from "plasticizers" like lipoamide to inhibitory peptides or targeted protein degraders 136, 137. Crucially, the loop is closed by the "Monitor" stage, where the initial diagnostic tools are re-employed to assess the therapeutic effect—such as a decrease in condensate viscosity or the disassembly of an oncogenic hub. This feedback allows for the dynamic adjustment of dosage or strategy, completing the personalized medicine loop and moving the treatment of condensatopathies towards a new standard of precision.

### 4.2 Diagnostics and Imaging: Visualizing the Aberrant Phase

**Figure 4:**
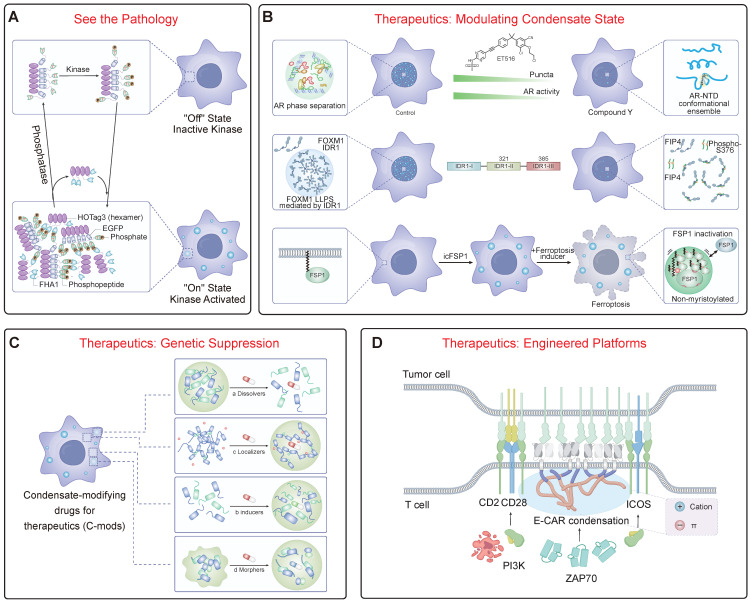
** Theranostic Strategies for Targeting Condensatopathies.** (A) Diagnostics: "See the Pathology." Illustrates genetically encoded reporters (e.g., SPARK) designed based on LLPS principles. Upon activation by an upstream pathogenic signal (e.g., a kinase), the diffuse reporter undergoes modification-dependent phase separation into fluorescent droplets, enabling the real-time visualization of aberrant signaling pathways. (B) Therapeutics: Modulating Condensate State. (Top) Disrupting pathological condensates. Small molecules (e.g., ET516) can bind to the androgen receptor (AR) to inhibit its LLPS and transcriptional activity. (Middle) Inhibitory peptides (e.g., FIP4) can target the IDR of an oncoprotein (FOXM1) to disrupt its condensate formation and inhibit tumor growth. (Bottom) Inducing therapeutic condensation. Conversely, specific small molecules (e.g., icFSP1) can induce the phase separation of a target protein (FSP1), "sequestering" it into non-functional condensates to inhibit its function and promote ferroptosis. (C) Therapeutics: Genetic Suppression. This approach targets the foundational scaffold of a pathological condensate. Antisense oligonucleotides (ASOs) can be designed to degrade a specific "scaffold" RNA (e.g., a repeat expansion RNA or lncRNA). By removing this core scaffold, the recruitment of client proteins is blocked, leading to the fundamental collapse of the entire pathological condensate. (D) Therapeutics: Engineered Platforms. Harnessing LLPS principles to engineer cellular therapies. For example, Chimeric Antigen Receptor (CAR)-T cells can be engineered with phase-separating motifs. This drives the formation of a highly organized, phase-separated "immunological synapse." This engineered condensate enhances T-cell activation and persistent tumor-killing efficacy.

A core tenet of theranostics is the ability to "see" the pathology (Figure 4A). The unique nature of aberrant condensates makes them ideal targets for molecular imaging. This can be achieved by visualizing the upstream signaling events that drive condensatopathies or by directly imaging the pathological assemblies. An innovative strategy for the former is the development of genetically encoded reporters that harness LLPS itself as a readout. For example, SPARK (Separation of Phases-based Activity Reporter of Kinase) reporters undergo phosphorylation-inducible phase separation, forming intensely fluorescent droplets in real-time upon activation of kinases like ATM, providing a direct window into the dysregulated signaling pathways that can trigger disease 102, 138. For directly imaging pathological structures, novel small-molecule probes are being developed. A prime example is TASG, a fluorogenic probe that selectively binds components within stress granules, allowing for the real-time tracking of their assembly and disassembly in live cells and *in vivo* 139. The unique composition of oncogenic and viral condensates also enables the design of highly specific imaging agents. On a broader scale, in oncology, gene expression signatures based on LLPS-related genes are being developed as powerful prognostic biomarkers to predict patient outcomes and response to immunotherapy in cancers like lung and bladder cancer, showcasing a new diagnostic paradigm 134, 135.

### 4.3 Therapeutic Interventions: Modulating, Degrading, and Restoring

Therapeutic strategies can be rationally designed to counteract the specific pathogenic mechanisms of LOF and TGOF. One major goal is to modulate material properties to prevent pathological LST. This can be achieved with small molecules that act as "plasticizers" to maintain liquidity, such as lipoamide, which dissolves SGs via redox modulation and shows benefit in ALS models 136. A second major strategy is to block the aberrant interactions that drive pathological condensation, an approach whose design is directly informed by the “molecular grammar” of phase separation (Figure 4B). This strategy is rooted in the “stickers-and-spacers” model described in Section 2.1. By developing small molecules or biologics that either competitively bind to key “stickers” (e.g., aromatic or charged residues) or sterically interfere with the interaction network, it is possible to precisely dismantle the multivalent forces holding a pathological condensate together. This principle has seen remarkable success in oncology. For instance, small molecules like ET516 specifically disrupt androgen receptor condensates to overcome antiandrogen resistance in prostate cancer 140, 141. The same logic applies to biologics; a rationally designed peptide targeting a key “sticker” region within the oncoprotein FOXM1 can disrupt its phase separation, thereby reducing breast tumor growth and metastasis 142. A revolutionary new approach involves using small molecules not to dissolve, but to induce a therapeutically beneficial phase separation. For instance, the small molecule icFSP1 triggers the condensation of FSP1, which sequesters it from the membrane and inhibits its anti-ferroptotic function, thereby promoting cancer cell death 143.

Expanding on the clinical translation of condensate-targeting strategies, several inhibitors have already advanced to clinical stages. First, the phosphatase SHP2 provides a paradigmatic example of targeting condensate-driven oncogenesis. Pathogenic SHP2 mutants have been shown to undergo aberrant phase separation, which hyperactivates the RAS-MAPK pathway. Allosteric inhibitors of SHP2, such as TNO155 and SHP099, act as "molecular glues" that lock the enzyme in a closed, auto-inhibited conformation. This conformational restriction prevents the multivalent interactions required for LLPS, thereby attenuating downstream signaling and overcoming drug resistance 144, 145.

Second, targeting the Hippo pathway effectors YAP/TAZ has yielded promising clinical candidates. YAP and TAZ form phase-separated nuclear condensates that compartmentalize the transcription factor TEAD and co-activators like BRD4 and MED1 to drive oncogenic gene expression 146, 147. The small-molecule inhibitor ETS-006 disrupts the YAP/TEAD interaction within these transcriptional hubs. Its rapid progression to clinical trials in both China and the U.S., along with FDA orphan drug designation, underscores the viability of dismantling oncogenic condensates.

Finally, the established cancer drug Olaparib (a PARP inhibitor) operates via a mechanism intrinsically linked to phase separation. Poly(ADP-ribose) (PAR) chains function as potent molecular seeds that trigger the liquid demixing of intrinsically disordered proteins (such as FUS and EWS) at sites of DNA damage. By inhibiting PAR synthesis, Olaparib prevents the nucleation of these repair condensates, thereby inhibiting efficient DNA repair and sensitizing tumor cells to DNA damage 148.

Targeted protein degradation (TPD) and genetic suppression offer powerful ways to eliminate the core components of pathological condensates (Figure 4C). A prime example of genetic suppression is the use of antisense oligonucleotides (ASOs). This approach directly addresses RNA's role as a master regulator and scaffold in condensate assembly, a concept detailed in Section 2.2. In many repeat expansion disorders and some cancers, a specific non-coding RNA acts as the foundational scaffold, initiating condensate formation through a pathogenic “Seed -> Bind -> Recruit” mechanism (Figure 1B). By using ASOs to target and degrade these specific RNAs, we can effectively remove the foundational “seed,” preventing the recruitment of client proteins and collapsing the entire pathological structure from its origin. This strategy has shown tremendous promise, with successful preclinical and clinical results for targeting key drivers like FUS and Ataxin-2 149-151. Alternatively, strategies can target proteins directly for degradation. Proteolysis-targeting chimeras (PROTACs) are heterobifunctional molecules that link a target protein (e.g., BRD4) to an E3 ubiquitin ligase, marking it for proteasomal degradation 152. A more advanced approach hijacks the cell's endogenous quality control hubs. For example, the PDF-Bin platform utilizes a bi-specific nanobody to physically bridge a protein of interest to p62 bodies, which function as versatile “degradation factories” capable of eliminating targets via both proteasomal and autophagic pathways 153. Another promising strategy is to target the enzymes that regulate condensates; for instance, inhibiting the deneddylase NEDP1 promotes SG disassembly and ameliorates ALS phenotypes in preclinical models 154.

### 4.4 Integrated "See-and-Treat" Theranostic Platforms

The ultimate goal is to merge these diagnostic and therapeutic modalities. Engineered cellular therapies are actively harnessing LLPS to create more potent therapeutics (Figure 4D). A prime example is the optimization of CAR-T cell therapy. By engineering CARs with phase-separating motifs, it is possible to drive the formation of a highly organized immunological synapse, leading to superior antigen sensitivity and persistent tumor-killing in preclinical models 50. A related concept is the design of "designer membraneless organelles" that can sequester specific endogenous proteins to control cell behavior. In this strategy, an engineered disordered protein scaffold is expressed to form synthetic condensates, which then recruit and functionally insulate native target proteins that have been tagged with a cognate high-affinity binding motif 155, 156. This platform provides a modular and reversible way to control cellular pathways, from cell cycle progression to cytoskeletal organization 155.

Another innovative strategy involves hijacking endogenous quality control systems. Forcing the proximity of aggregation-prone TDP-43 to the PML nuclear body PQC hub was sufficient to trigger a protective SUMOylation-ubiquitylation cascade that shielded TDP-43 from aggregation 98. This concept extends to nuclear import receptors (NIRs), which act as chaperones to dissolve aberrant RBP phases; enhancing NIR activity is a promising therapeutic avenue 157, 158. Further building on this, a highly modular platform using bi-specific nanobodies has been developed to bridge any protein of interest to p62 bodies for degradation, creating a universal “degradation factory” 153. These sophisticated strategies, which move beyond simple inhibition to actively remodel cellular organization, represent the future of therapies for condensatopathies.

### 4.5 Therapeutic Decondensation: Lessons from Pioneer Factors

Beyond modulating material properties or blocking interactions, an emerging therapeutic concept involves the active dissolution or unpacking of aberrant condensates. Nature provides a blueprint for this process through the action of pioneer transcription factors, which must engage and open condensed, silent chromatin to establish new gene expression programs. A key example is the pioneer factor FOXA1, whose function in development and cancer is mechanistically coupled to its ability to remodel condensates. A detailed study revealed that FOXA1 utilizes its intrinsically disordered N- and C-terminal IDRs to form dynamic, submicron-sized condensates in the nucleus. These are not static structures; rather, they function as active agents that can invade and dissolve pre-existing condensed chromatin domains, such as those compacted by linker histone H1. This represents a "phase separation transition," where FOXA1 condensates actively unpack heterochromatin to create locally accessible euchromatin 159. This mechanism explains how FOXA1 can function as a pioneer factor and suggests a novel therapeutic principle: designing molecules that mimic the condensate-dissolving properties of pioneer factors could offer a powerful strategy to deconstruct pathological condensates, reverse epigenetic silencing, and restore normal cellular function.

Inspired by these natural decondensation principles, a new frontier of "reverse engineering" is emerging, aimed at creating programmable therapeutic tools. If the "molecular grammar" of promoting IDRs (pIDRs) is known, a key question arises: does an "anti-grammar" exist for IDRs that actively inhibit or dissolve condensates? The discovery and characterization of such inhibitory IDRs (iIDRs) could lead to the development of a novel class of modular biologics. By fusing these iIDR domains—acting as potent "dissolving warheads"—to targeting modules like nanobodies, it may become possible to create "molecular scalpels" that can be programmed to precisely locate and dismantle specific pathological condensates, including solid-like aggregates previously considered "undruggable." This synthetic biology approach, moving from modulating to actively engineering the dissolution of condensates, represents a bold and promising direction for future therapeutic design.

## 5. Conclusion and Perspective

The recognition of LLPS as a fundamental principle of cellular organization has profoundly reshaped our understanding of biology and disease 14. The field is now moving beyond a simple liquid-equals-functional, solid-equals-pathological dichotomy, embracing the concept of “tunable metastability” to reconcile the dual roles of condensates 46. This paradigm shifts the therapeutic goal from merely dissolving aggregates to a more sophisticated strategy of “condensate state modulation.” However, to translate this fundamental knowledge into clinical solutions, the field must systematically confront three core bottlenecks.

First is the discovery bottleneck: the landscape of disease-relevant condensates remains incomplete, with research heavily focused on a few "star proteins." Overcoming this requires a new paradigm of systematic discovery, integrating AI-driven prediction with high-throughput multi-omics and functional screening to systematically map the full "condensatome" of human disease. Second is the mechanism bottleneck: the causal chain from upstream signaling to the downstream functional consequences of aberrant phase transitions is often broken by a lack of structural evidence. Bridging this gap demands the full power of *in situ* cryo-electron tomography (cryo-ET) to capture the high-resolution "structural fingerprint" of pathological condensates within their native cellular context, providing the ultimate "seeing-is-believing" proof.

Third, and perhaps most critically, is the intervention bottleneck: a lack of therapeutic platforms designed to specifically target the physical *state* of condensates. However, this approach faces significant challenges, primarily specificity. Given that the "molecular grammar" (e.g., cation-pi interactions) is shared across many physiological condensates, designing "c-mods" that target only the pathological assemblies without disrupting essential organelles remains a formidable hurdle. The future lies in creating programmable, broadly applicable technologies. This includes not only refining "condensate-modifying drugs" (c-mods) but also pioneering "reverse-engineered" tools, such as iIDRs acting as "molecular scalpels" to precisely dismantle aberrant assemblies. Moving from theory to practice, emerging evidence suggests that this can be realized using engineered peptides or fusion tags designed with a specific "anti-grammar"—characterized by high-density negative charges. These polyanionic modules can actively infiltrate and electrostatically disrupt the interaction network of pathological, cationic condensates, offering a programmable strategy to reverse even solid-like aggregated states. Ultimately, the goal is to establish a "discover-resolve-intervene-rediscover" research loop, where novel intervention tools serve not only as therapies but also as probes to reveal deeper mechanistic insights and uncover new targets.

Furthermore, a critical translational hurdle lies in the "evolutionary conservation gap." While the "molecular grammar" of phase separation is conserved, the specific sequences of IDRs can vary significantly between humans and model organisms like mice. Consequently, the phase behavior and drug response of condensates in animal models may not always perfectly recapitulate human pathology, necessitating rigorous validation in human-derived systems (e.g., iPSC models).

By tackling these bottlenecks, the reach of condensatopathies will undoubtedly extend beyond neurodegeneration and cancer, offering new explanations for enigmatic processes in immunology, memory formation, and aging. Integrating the physics of tunable metastability with advanced chemical biology, *in situ* structural analysis, and synthetic engineering, the study of biomolecular condensates is poised to become a central and transformative pillar of 21st-century precision medicine.

## Figures and Tables

**Table 1:** Key Molecular Players in Condensate Biology

Category	Molecule / Complex	Key Features & Function in Phase Separation	References
Core Scaffolds & RBPs	FUS, TDP-43, TAF15, hnRNPA1/A2	PrLD-containing proteins; drivers of physiological LLPS. Pathogenic mutations accelerate liquid-to-solid transition (LST), forming amyloid precursors in ALS/FTD.	17, 37
	G3BP1/2, TIA-1, CAPRIN1	Nucleators of Stress Granules (SGs); assembly driven by IDRs and regulated by ionic stress. G3BP1 is protective in Huntington's disease.	101
	NPM1, Fibrillarin (FBL)	Scaffold proteins of the nucleolus (GC and DFC compartments). NPM1 is a target of C9orf72 DPRs; mutations drive AML.	122, 160
Transcriptional & Chromatin Regulators	Mediator (MED1), BRD4, Pol II CTD	Form transcriptional hubs at super-enhancers to concentrate machinery; regulated by phosphorylation.	161
	Fusion Oncoproteins (e.g., NUP98-HOXA9, FUS-CHOP)	Drive formation of aberrant "oncogenic condensates" that hijack transcriptional machinery in leukemia and sarcoma.	126
	FOXA1, p53, MeCP2	Pioneer factors and tumor suppressors that utilize LLPS to unpack chromatin (FOXA1) or activate targets (p53). MeCP2 compacts heterochromatin.	59, 159, 162
	HP1α (Swi6)	Drives heterochromatin condensation, essential for nuclear stiffness and 3D genome organization.	58, 163
Pathological & Viral Drivers	Tau, α-synuclein, Huntingtin (HTT)	Undergo pathological LST from liquid droplets to amyloid aggregates; can co-phase separate.	123, 164
	C9orf72 DPRs	Toxic dipeptide repeats that disrupt nucleocytoplasmic transport and infiltrate the nucleolus.	124, 165
	Viral Proteins (e.g., N protein, nsP3)	Hijack host machinery (e.g., G3BP) to form viral replication factories or disrupt host immune condensates.	166
Quality Control & Signaling	Chaperones (Hsp90, Hsp70, HSPB1, YAP)	Maintain "granulostasis" by binding IDRs to preserve fluidity and prevent aggregation. YAP acts as a non-canonical chaperone for TDP-43.	20, 21, 64
	PQC Hubs (PML, p62/SQSTM1)	Sequestration and degradation centers. PML bodies facilitate SUMO-ubiquitin modifications; p62 condensates target cargo for autophagy.	98, 167
	Engineered CARs	Synthetic receptors with phase-separating motifs (e.g., CD3ε) that enhance immunological synapse formation.	50

**Table 2:** Regulation of Condensate Homeostasis

Regulatory Mechanism	Key Modulator & Example	Mechanism of Action	References
Post-Translational Modifications (PTMs)	Phosphorylation (DYRK3, GSK3β)	Acts as a "dissolvase" switch (DYRK3) during mitosis or catalyzes aggregation (GSK3β on Tau).	168
	Ubiquitination / SUMOylation (TRIM21, RNF4)	Signals for disassembly or recruits clients to PQC hubs (e.g., PML bodies).	169
	Methylation (PRMTs) / Acetylation	Modulates cation-π interactions (Arginine methylation) or charge (Acetylation) to tune condensate specificity and phase behavior.	170, 171
	PARylation (PARPs)	Poly(ADP-ribose) acts as a potent scaffold to seed condensation (e.g., FUS at DNA damage sites) or promote toxicity.	172
Environmental Cues	Physicochemical (Temp, pH, Ions)	Direct sensing of temperature (HSF1), pH (His-rich IDRs), or ionic strength (Na⁺, Zn²⁺) triggers adaptive phase separation.	83, 173
	Metabolic & Mechanical (ATP, Stiffness)	ATP acts as a hydrotrope to prevent aggregation. Mechanical force triggers LLPS of sensors like YAP/TAZ.	89, 90
Quality Control Systems	RNA Chaperones (DDX Helicases)	Use ATP hydrolysis to remodel RNA networks and maintain condensate fluidity.	80
	Granulophagy (p62, NCOA7)	Selective autophagy pathway for clearing terminally damaged condensates.	174

**Table 3:** The Spectrum of Condensatopathies

Disease Category	Disease & Key Drivers	Core Pathogenic Mechanism (LOF / TGOF)	Theranostic Approaches	Refs
Neurodegeneration	ALS / FTD (TDP-43, FUS, C9orf72)	Double-Hit: TGOF (toxic aggregation) + LOF (nuclear depletion). Accelerated by aging and PQC decline.	See: NfL, PET. Treat: ASOs (ION363), c-mods (lipoamide), PQC activation.	37, 175
	Alzheimer's / Parkinson's (Tau, α-syn)	TGOF: Pathological LST to amyloid. LOF: Impaired chromatin interaction or P-body function.	See: PET, RT-QuIC. Treat: Aggregation inhibitors, antibodies.	38, 123
Cancer	Solid Tumors (YAP/TAZ, AR-V7)	TGOF: "Oncogenic condensates" at super-enhancers drive overexpression and drug resistance.	See: LLPS signatures. Treat: Hub disruption (BRD4i, ET516), covalent inhibitors.	89, 140
	Fusion Malignancies (NUP98-HOXA9)	TGOF: Fusion proteins form aberrant nuclear hubs to rewire transcriptional programs.	See: Cytogenetics. Treat: PQC remodeling (ATRA/Arsenic).	176
Cardio-Metabolic	Cardiomyopathy / Fibrosis (RBM20, VGLL3)	LOF/TGOF: Dysregulated LLPS drives pathological remodeling and fibrosis, exacerbated by aging.	See: Imaging. Treat: Gene editing, targeting mechanosensors.	30, 177
	Diabetes / Cataracts (IAPP, α-crystallin)	TGOF: Metabolic stress drives amyloid gelation (IAPP) or aggregation (crystallin).	See: Clinical exams. Treat: Aggregation inhibitors, small-molecule chaperones.	178
Developmental	Rett Syndrome (MeCP2)	LOF: Mutations impair MeCP2-mediated chromatin condensation, leading to heterochromatin defects.	See: Sequencing. Treat: Gene therapy, chromatin restorers.	59
	Infertility / Sensory (Cdh23, CCER1)	LOF: Defects in assembly of structural condensates in germ cells or sensory organs.	See: Clinical testing. Treat: Gene therapy.	179
Infectious	Viral Infections (SARS-CoV-2, HIV)	TGOF (Hijacking): Viruses form replication factories and disrupt host antiviral condensates.	See: PCR. Treat: Disruptors (e.g., GCG), host factor targeting.	180

**Table 4:** Theranostic Strategies Targeting Aberrant Condensates

Strategy Category	Therapeutic Approach	Mechanism & Example Target	Refs
Small Molecule Modulators	Dissolvers / Plasticizers	Alter physical properties to restore liquidity (e.g., Lipoamide for SGs).	181
	Inhibitors of Oncogenic Hubs	Block formation of transcriptional condensates (e.g., ET516 for AR; ETS-006 for YAP/TEAD).	147, 182
	Molecular Glues	Lock proteins in closed states to prevent LLPS (e.g., TNO155 for SHP2).	144
	Condensate Inducers	Trigger protective or toxic condensation (e.g., icFSP1 for ferroptosis; Arsenic Trioxide).	143, 183
Genetic & Protein Engineering	Antisense Oligonucleotides (ASOs)	Degrade scaffolding RNAs to collapse condensates (e.g., ION363 for FUS).	149
	Targeted Degradation (PROTACs)	Eliminate core scaffold proteins (e.g., BRD4 degraders).	152
	Engineered Therapies	Use LLPS to enhance function (e.g., Phase-separated CAR-T) or target degradation (PDF-Bin).	50, 153
Diagnostics	Imaging Probes & Signatures	Fluorogenic probes for viscosity/aggregation (TASG, Molecular Rotors) or gene signatures for prognosis.	133, 139

## Abbreviations

ALS: amyotrophic lateral sclerosis
AML: acute myeloid leukemia
ASO: antisense oligonucleotide
CAP: chromatin-associated protein
CAR: chimeric antigen receptor
c-mod: condensate modifying drug
cryo-EM: cryo-electron microscopy
Csat: saturation concentration
DFC: dense fibrillar component
DLB: dementia with Lewy bodies
DPR: dipeptide repeat
FTD: frontotemporal dementia
GC: granular component
HTT: Huntingtin
IDP: intrinsically disordered protein
IDR: intrinsically disordered region
LC: low complexity
LCD: low-complexity domain
LLPS: liquid-liquid phase separation
LOF: loss-of-function
LST: liquid-to-solid transition
m1A: N1-methyladenosine
m6A: N6-methyladenosine
MBD: methyl-binding domain
MLO: membraneless organelle
MSP: multisystem proteinopathy
ncRNA: non-coding RNA
NF2: neurofibromatosis type 2
NIR: nuclear import receptor
PD: Parkinson's disease
PE: prime editor
PQC: protein quality control
PrLD: prion-like domain
PROTAC: proteolysis-targeting chimera
PTM: post-translational modification
RBP: RNA-binding protein
RNP: ribonucleoprotein
RTT: Rett syndrome
SG: stress granule
sHSP: small heat shock protein
TGOF: toxic gain-of-function
TPD: targeted protein degradation
TRD: transcriptional repression domain
